# Complex assessment of immunosuppression effects in prevention and treatment of adhesive disease, an experiment

**DOI:** 10.25122/jml-2021-0371

**Published:** 2022-06

**Authors:** Dauren Auzhanov, Meirbek Aimagambetov, Nazarbek Omarov

**Affiliations:** 1 Department of Hospital Surgery, Non-Commercial Joint-Stock Company Semey Medical University, Semey, Kazakhstan

**Keywords:** postoperative period, adhesive disease, scars, azathioprine, blood changes, CG – control group, EG – experimental group, PI – phagocytic index

## Abstract

The cause of all small bowel obstruction in 60–75% of cases is adhesive development. The first and main method for adhesion prevention is undoubtedly the surgical technique, but the prevention of adhesive development is still actual. We aimed to study macroscopic and microscopic peculiarities of the intestine, peritoneum, and scars of the anterolateral abdominal wall. Also, immunological blood changes were observed in rats with the experimental created adhesive disease on the background of azathioprine introduction. The experiment was conducted on 40 rats divided into 2 subgroups: 20 animals as an experimental group (EG1) and 20 as a control group (CG1). Animals from EG received azathioprine (Moshimerampreparaty named by N.A. Semashko, Russia) in a dosage of 1 mg/100g of weight once a day for the first 3 days (starting from the day of surgery). The control group did not receive any drugs. All 40 rats survived the postoperative period. Rats were removed from the experiment on the 7^th^ day after the operation. There were significant statistical differences in most indicators between the experimental and control groups. Phagocytic index (PI) was reduced by 4.55 due to the natural reaction of the rat organism to the surgery. Indicators of EG were a slight decrease in leukocytes and lymphocytes by 0.3 and 0.9, respectively, a moderate decrease in T-lymphocytes by no more than 2.0, and a decrease in phagocytic activity by 5.8. Immunosuppression with azathioprine significantly reduced the frequency and severity of the adhesive process of the abdominal cavity. Used in the recommended dose does not significantly inhibit important indicators of immunity and does not affect wound healing processes.

## INTRODUCTION

The development of adhesive disease after abdominal surgery has a pronounced negative impact on patients' conditions and the healthcare systems. The adhesions of the peritoneal cavity are a physiological response to surgical trauma. Usually, this process does not have a pathological effect after surgery. However, there are some exclusions. Adhesions can lead to adhesive intestinal obstruction and acute surgical disease if some predisposing factors are present. Moreover, it can become a chronic disease (adhesive disease) [1, 2]. The cause of all small bowel obstructions in 60–75% is adhesive development [1, 3]. The development of adhesive disease with clinical symptoms can reach 80% and cause more than 20% of emergency surgery for abdominal pain [4, 5]. Adhesions of the small pelvis can cause infertility in 10% and chronic pelvic pain for 25% of women [6]. There is a risk of complications in patients with adhesions, and in 95% of cases, secondary laparotomy has technical difficulties [6–8]. Consequently, hospital treatments become more expensive. For example, the total annual cost of treating adhesion-related complications in the United States alone exceeds $2 billion [3, 9].

The first and primary method for adhesion prevention is certainly a surgical technique, but looking for therapeutic ways to prevent the adhesion development is still actual [1, 10]. A significant decrease in the intra-peritoneal adhesions was observed when using hyaluronic acid film, but there was no evidence of decreasing postoperative intestinal obstruction. Moreover, abdominal abscess and intestinal anastomosis failure were identified [11–13]. Using icodextrin had no significant impact on decreasing the reoperations frequency in adhesive intestinal obstruction [14]. Solid barriers have several drawbacks, like the liquid barriers [11]. Using glucocorticosteroids, non-steroidal anti-inflammatory drugs, antihistamines, and intraperitoneal injection of antibiotics has no proven effect [15, 16]. Temporary controlled immunosuppression is of interest nowadays. The anti-adhesion effect of immunosuppressants (5-fluorouracil and cyclophosphamide) was noted in several studies [17, 18] due to leucocyte function suppression. However, the disadvantage of these drugs was a pronounced cytotoxic effect and the input route: intraperitoneal and endolymphatic.

The traditional conservative treatment is based on decompression of the gastrointestinal tract, spasm relief, stimulation of peristalsis, intra-intestinal administration of water-soluble contrast, and antibiotics [1, 19–21]. Unfortunately, the complexity of conservative treatment methods is not always effective, and more radical treatment methods are often required. Surgical treatment (adhesiolysis) is indicated for: complications of the adhesive process (acute intestinal obstruction), ineffective conservative therapy for partial intestinal obstruction, the treatment of infertility, and the elimination of chronic pain [22]. However, the choice of surgical and non-surgical tactics for the management of patients requires thoughtful decision-making, and many factors must be taken into account [23].

This study aimed to investigate macro-and microscopic peculiarities of the intestine, peritoneum, scars of the anterolateral abdominal wall, and immunological blood changes in rats with experimental adhesive disease under azathioprine administration.

## MATERIAL AND METHODS

The study was conducted in the educational and research laboratory of the non-commercial joint-stock company Semey Medical University, Republic of Kazakhstan.

6 rats were used for a preliminary assessment of laboratory parameters and anatomy of internal organs prior to the experiment. Laboratory indicators of this group did not differ from the average norm and were taken as the norm. The main experiment was conducted on 40 adult (4–5 months) male Wistar rats weighing 200±20 g (M±σ). Animals were kept under standard conditions in a climate-controlled room (relative humidity 40±5%, room temperature 21–24°C), and a 12-hour lighting cycle (light/dark). Before and after surgical procedures, food and water were made available ad libitum. All surgical procedures were performed under general anesthesia with ketamine preparations (FISIOPHARMA SRL, ITALY) at a dose of 80 mg/kg body weight and 5 mg/kg xylazine (NITA-PHARM, RUSSIA). Animals were contained in standard vivarium conditions according to the "European Convention for the protection of vertebrate animals used for experimental and other scientific purposes" (Strasbourg, 18.03.86 G.) All manipulations of animals were carried out in compliance with the rules of humane treatment of laboratory animals and the use and maintenance of laboratory animals (Order No. 755 of the USSR Ministry of Health of 07/12/1977), as well as considering the requirements and conditions set out in the Declaration of Helsinki by the World Medical Association. The animals were withdrawn from the experiment by overdosing on anesthetics, considering the provisions regulated by order No. 724 of 1984, "Rules for working with experimental animals" (Ministry of Higher Education, Union of Soviet Socialist Republics).

### Experiment design

The Blur operation was performed on 40 animals to induce an adhesion process in the abdominal cavity: median laparotomy (2.0–3.0 cm), the intestines and peritoneum were dusted with sterile talc. The talc was evenly dispersed on the surface of the parietal and visceral sheets of the peritoneum. The role of talcum powder in the induction of the adhesion process is so evident that it has become one of the main methods of modeling intra-abdominal adhesions in animal experiments [24]. The laparotomic wound was sutured with interrupted sutures (Vikril 4.0 thread (ETHICON, USA). The duration of each operation was 10±3 minutes. After the operation, the animals were kept under an infrared lamp until they were awakened from anesthesia.

The rats were divided into 2 subgroups: 20 animals as an experimental group (EG1) and 20 as a control group (CG1). Animals from EG received the drug azathioprine (Moshimerampreparaty, named by N.A. Semashko, Russia) in a dosage of 1 mg/100 g of weight once a day for the first 3 days (starting from the day of surgery). The drug was mixed with drinking water and given orally using a syringe. Control group rats did not receive any drugs. All 40 rats survived the postoperative period. Rats were removed from the experiment on the 7^th^ day.

Blood from the carotid arteries was sent for immunological analysis. Furthermore, the peritoneal cavity was opened, and a visual macroscopic assessment of the adhesion process was performed. Then, adhesions with areas of the involved organs were sent for histological examination. A clinical assessment of the state of postoperative wounds was scored after 7 days; after opening, the scars were excised and subjected to microscopic evaluation.

### Macroscopic evaluation

We conducted a visual evaluation of the adhesions degree after opening the abdominal cavity of all animals in the EG and CG groups. Quantitative assessment of adhesions was scored on the Moreno scale, and qualitative assessment on our modified Binda scale [25]. Wound healing was assessed (condition of the wound edges, swelling, hyperemia, the presence of detachable or diverging wound edges) for 7 days.

### Histological (microscopic) assessment

The histological material (small intestine at the place of adhesion, skin with postoperative scars) underwent fixation in 10% neutral formaldehyde. Serial histological sections of 5 µm thickness after dewaxing were stained with hematoxylin and eosin, and according to Van Gieson, an assessment was performed on vascular proliferation, inflammation, fibrosis, and collagen formation. Histological evaluation was performed using the assessment system by Hooker et al. [26] and described in Table 1. The degree of leukocyte infiltration, the number of fibroblasts, and loose connective tissue in scars were evaluated.

**Table 1:** Evaluation scale for fibrosis and inflammation.

Description	Points
**Fibrosis**	
No	0
Minimal, loose	1
Middle	2
Pronounced, dense	3
**Inflamatory**	
No	0
Giant cells, random scattered lymphocytes and plasma cells	1
Giant cells with an increased number of mixed lymphocytes, plasma cells, eosinophils, neutrophils	2
A lot of mixed inflammatory cells, microabscesses	3

### Immunological analysis

The leukocyte formula was created for each animal. Moreover, we studied the phagocytic index by considering the total number of blood phagocytes. Surface immunofluorescence reaction using a set of unconjugated monoclonal antibodies (CALTAG Laboratories) to determine the total number of lymphocytes and subpopulations (CD3+, CD4+, CD8+) was used.

### Statistics

The statistical package SPSS (version 22) was used for data processing. The research results are presented as absolute numbers and medians with quartiles Me (Q1, Q3). Kolmogorov-Smirnov criterion was used to check for normal distribution in comparison groups. The criteria of Mann-Whitney, Fisher's exact test (F), Pearson χ2 test, and Yates correction χ2 test were used in the calculations, and a p-value<0.05 was considered statistically significant.

## RESULTS

### Impact on adhesion formation

The results of the first group showed that the adhesion process was observed in 100% (20 rats) in CG; and in 70% (14 rats) in EG1 F-0,01010, p<0.05. In CG, the prevalence of formed adhesions was 50 to 15, U=5.0, Z=-5.480, p<0.001, and the thickness of the adhesions and their strength (tenacity) were significantly higher, p<0.05 (Table 2).

**Table 2:** Moreno scale. Quantification of adhesion formation in rat groups.

Criteria	Control group 1, n=20	Experimental group 1, n=20	U	Z	R
**Total number**	50	15	5.0	-5.480	<0.001
			**χ2**	**df**	**R**
**Type of adhesions**					
**Parietal** **Visceral**	31 19	13 2	19.333 19.976	2 2	<0.001 <0.001
**Thickness**					
**>3mm** **<3mm**	31 19	3 12	25.342 4.606	2 2	<0.001 0.1
**Tenacity**					
**Type 0** **Type 1** **Type 2** **Type 3**	- 12 17 21	- 13 2 -	- 3.720 15.231 29.565	- 2 2 2	- 0.156 <0.001 <0.001

The prevalence of the adhesion process was fixed in CG. The adhesion process covered a significantly larger volume of areas of the peritoneal cavity: in 3 animals within 1 region (in EG – 14), 2 regions – in 10 cases, and the total adhesion process in 7 animals (Table 3). In EG, adhesions in the abdominal cavity were completely absent in 6 animals.

**Table 3:** Binda scale.

Degree of adhesion	Points	Extent (animals number)		Type of adhesions (adhesions number)		Tenacity (adhesions number)	
		CG (n=20)	EG (n=20)	CG (n=50)	EG (n=15)	CG (n=50)	EG (n=15)
**Easy**	0	0	6	-	3	-	3
	1	3	14	2	13	12	13
**Pronounced**	2	10	-	21	2	17	2
	3	7	-	27	-	21	-
		χ2-26.189; df-1, p<0.001		F-0.00000, p<0.05		χ2-20; 407; df-1, p<0.001	

χ^2^ – chi-test with Yates correction; F – exact Fisher Criterion.

According to their structure, the adhesions formed in CG were dense and vascularized; the separation or the use of the acute method was required for separation. In EG, adhesions of the film structure were easily disconnected even with little effort.

We analyzed the adhesive process and divided it into easy and pronounced according to the severity. The CG had substantially more revelation of the pronounced adhesive process. There were significant differences (p<0.05 and p<0.001) in the three criteria of the control and the experimental group according to the severity of the adhesive process.

We performed a histological assessment to compare the degree of fibrosis and inflammation: easy (0–1 points) and pronounced (2–3 points) (Table 4). The pronounced degree of fibrosis in CG significantly prevailed than in EG1 χ2=4.949, df=1, p=0.027. According to the signs of inflammation, no significant difference was detected χ2=0.909, df=1, p=0.341.

**Table 4:** The degree of fibrosis and inflammation.

Points	CG (n=20)	EG (n=20)	χ2	df	R
**Fibrosis**					
0	0	3	4.949	1	0.027
1	5	10			
**Easy**	5	13			
2	10	7			
3	5	0			
**Pronounced**	15	7			
**Inflammation**					
0	1	3	0.909	1	0.341
1	9	10			
**Easy**	9	13			
2	10	7			
3	1	0			
**Pronounced**	11	7			

χ^2^ – Yates correction chi-test.

### Effect on the healing of postoperative wounds

The clinical evaluation of the postoperative wounds on the 4^th^ day revealed that in CG, there was moderate edema and slight hyperemia of the wound edges in 60% (12 rats) and 40% (8 rats) in EG1 cases (p=0.206). On the 7^th^ day, in both groups, the wounds were clean; there was no edema and hyperemia in the phase of initial epithelization.

### Microscopy study

Microscopy was conducted on the 7^th^ day. Pronounced signs of leukocyte infiltration were not fixed in EG and CG groups. The moderately expressed inflammatory reaction was detected in 25% of rats in the CG group (5 animals), and for one rat in the EG group (5%). Weakly expressed reaction was fixed in 5 animals in the CG group (25%), and for the EG group, this characteristic was lower (2 rats – 10%). In both groups, CG and EG, mononuclear histiocytes (initial signs of repair) were observed in 6 and 5 cases, respectively. Fibroblasts were fixed rare cells. The post-surgery wound was healing by primary intention.

### Influence on immunity indicators

Laboratory tests of some indicators of immunity were determined in 15 animals of each subgroup (Table 5 and Table 6). The tables show significant statistical differences in most indicators between the three groups. Some indicators of the immune response are higher than normal (by 2.3–5.7) in the CG group. Phagocytic index (PI) was reduced by 4.55 due to the natural reaction of the rat organism to the surgery. The indicators in the EG group were a slight decrease in leukocytes and lymphocytes by 0.3 and 0.9, respectively, a moderate decrease in T-lymphocytes by no more than 2.0, and a decrease in phagocytic activity by 5.8.

**Table 5:** Indicators of the immune status of rats.

Indicators	Norm (Me)	CG. (Me)	EG. (Me)	U C.G.* E.G.	U C.G.* Norm	U EG* Norm
Leucocytes (*10^9^/L)	9.05	11.2 (10.8; 12; 1)	8.7 (8.6; 9.0)	0, Z=-4.674 p<0.001	0, Z=-3.507 p<0.001	15.5, Z=-2.315 p=0.021
**Lymphocytes (%)**	78.3	81.5 (80; 6; 82; 1)	77.4 (76.8; 77.8)	0, Z=-4.671 p<0.001	0, Z=-3.510 p<0.001	1.0, Z=-3.442 p=0.01
**CD3 (%)**	72.35	78.1 (77; 4; 78.4)	70.2 (70.0; 71.3)	0, Z=-4.671 p<0.001	0, Z=-3.512 p<0.001	8.0, Z=-2.891 p=0.04
**CD4 (%)**	43.2	48.1 (47.8; 48.7)	43.2 (42.8; 44.0)	0, Z=-4.673 p<0.001	0, Z=-3.516 p <0.001	31, Z=-1,093 p=0.274
**CD8 (%)**	28.7	26.5 (25.9; 27.1)	26.7 (25.6; 27.1)	104, Z=-0.353 p=0.724	3.5, Z=-3.243 p=0.001	3.5, Z=-3.248 p=0.01
**Phagocytic index (PI)**	91.5	87.0 (86.0; 88.0)	86.0 (84.0; 87.0)	62.5, Z=-2.111 p=0.035	0, Z=-3.539 p<0.001	0, Z=-3.531 p<0.001
**Phagocytic number (PN.)**	4.15	4.2 (4.0; 4.2)	4.1 (4.0; 4.1)	78, Z=-1.473 p=0.141	43.5, Z=-0.121 p=0.903	27.5, Z=-1.428 p=0.153

**Table 6:** Hematological tests results.

Indicators	Me1-Me2 (C.G.* E.G.)	Me1-Me2 (C.G.* Norm)	Me1-Me2 (E.G.* Norm)
**Leucocytes**	2.5 CI 95% [2.2; 3.2]	2.3 CI 95% [1.8; 3.1]	0.3 CI 95% [0.1; 0.5]
**Lymphocytes**	4.3 CI 95% [3.5; 5.0]	3.2 CI 95% [2.3; 3.9]	0.9 CI 95% [0.4; 1.7]
**CD3**	7.7 CI 95% [6.8; 8.2]	5.7 CI 95% [5.1; 6.2]	2.0 CI 95% [1.0; 2.4]
**CD4**	4.8 CI 95% [4.2; 5.4]	5.1 CI 95% [4.7; 6.0]	0.4 CI 95% [-0.2; 1.2]
**CD8**	0.1 CI 95% [-0.6; 1.2]	2.05 CI 95% [1.5; 2.8]	2.0 CI 95% [1.6; 3.1]
**Phagocytic index**	1.0 CI 95% [0; 2]	4.55 CI 95% [3.5; 5.8]	5.8 CI 95% [4.6; 7.3]
**Phagocytic number**	0.1 CI 95% [0; 0.2]	0 CI 95% [-0.1; 0.1]	0.1 CI 95% [0; 0.2]

## DISCUSSION

The process of adhesion formation is a protective physiological response of the body to injury [27]. The immune system triggers a number of mechanisms, the main role belonging to cellular immunity (T-lymphocytes, macrophages). The matrix laying period for future adhesions proceeds mainly in the first three days after surgery [4, 27]. The impact on the functions of protective cells, as well as on inflammatory mediators in this period, can disrupt adhesion processes [27].

The basic principle of applying barrier techniques is the separation of damaged sections of the peritoneum and internal organs. The method is effective, mainly at the place of application. So, it is expensive because it requires considerable resources, especially for large damaged areas [1, 11].

Using immunosuppression to prevent the evolution of postoperative adhesions is of great interest in connection with the impact of these drugs on the immunological elements of the pathogenesis of adhesions [17, 18]. Nevertheless, the routes of administration of these drugs are somewhat complex – endolymphatic and intraperitoneal. These drugs have a number of side effects in the near and distant time. This limits the study of immunosuppression as a prevention method for adhesions [27].

In our research, the anti-adhesion effect of azathioprine was tested. We conducted the experiment to study the effect of the immunosuppressive drug azathioprine on the primary processes of adhesion formation and the mechanism of action in the complicated course of adhesive disease (modeling of obstruction). The effect of the drug on some indicators of immunity and regeneration process was also studied. This drug has a less pronounced cytostatic effect and a more pronounced effect on cellular immunity. The oral input is simpler, and the short use period has no significant negative effect on the immune system and regeneration processes.

Using immunosuppression drugs to prevent adhesions should not be common and primary. An important role belongs to the primary state of the immune system, concomitant conditions of the body, and etiological factors, which lead to trauma to the peritoneal cover of the abdominal cavity. Contraindications to this technique are, of course, primary immunodeficiencies and severe systemic and intra-abdominal infections (peritonitis). However, the use of the technique with good sanitation and antibiotic therapy in acute non-purulent peritonitis can be further studied. We believe that the main indications for immunosuppressive anti-adhesion therapy in the absence of absolute contraindications can be extensive abdominal surgery, as well as the prevention of re-formation of adhesions after adhesiolysis.

Traditional conservative treatment regimens are aimed at improving intestinal motor function. A frequent inefficiency of traditional conservative methods is the disrupted delivery of the drug to the affected areas of the intestine. Severe tissue edema, impaired microcirculation, and capillary thrombosis close the drug's access to the point of application. Drugs do not penetrate or penetrate in insufficient quantities through the "inflammatory" barrier, leading to therapy ineffectiveness. Immunosuppression leads to the suppression of cellular immunity. A quick and powerful effect on inflammatory processes will improve the delivery of drugs to the desired point and reduce tissue swelling, which will lead to an improvement in the patency of the intestinal tube.

## CONCLUSION

Immunosuppression with azathioprine significantly reduces the frequency and severity of the adhesive process of the abdominal cavity. The recommended dose does not significantly inhibit important indicators of immunity and does not affect wound healing processes. By reducing the swelling of the intestinal wall, it is possible to improve the mechanical patency of the intestinal tube. The use of immunosuppression for the prevention and treatment of adhesive disease requires further examinations in clinical studies and the creation of clear algorithms for use.
